# Adolescent idiopathic scoliosis: current concepts on neurological and muscular etiologies

**DOI:** 10.1186/s13013-016-0066-y

**Published:** 2016-06-27

**Authors:** Marcelo Wajchenberg, Nelson Astur, Michel Kanas, Délio Eulálio Martins

**Affiliations:** Universidade Federal de São Paulo/UNIFESP, São Paulo, Brazil; Santa Casa de Misericórdia de São Paulo, São Paulo, Brazil; Hospital Israelita Albert Einstein, Av. Albert Einstein, 627, Pavilhão Vicky e Joseph Safra-3 andar/306, Morumbi, São Paulo 05651-901 Brazil

**Keywords:** Genetics, Scoliosis/etiology, Adolescent

## Abstract

Adolescent idiopathic scoliosis (AIS) is a frequent disease but its etiology remains unknown. Gender prevalence in females is already known and there are many suggested hypotheses to explain its origin and manifestation, like associated neurologic, muscular and connective tissue disorders. Literature reports have tried to analyze disease prevalence in selected populations, possible ways of inheritance, related genes location and their polymorphisms, which may play a role in the development of the deformity. The purpose of this paper is to review and update concepts on the origin and genetic influence on AIS.

## Background

Adolescent idiopathic scoliosis (AIS) is defined as a lateral deviation of the spine, associated to rotation of the vertebrae in an otherwise healthy subject, without any known cause for the deformity. These subjects have no neurological, muscular disturbance or any other diseases [1]. Radiographic imaging studies show no vertebral abnormalities but a spinal curve of greater than 10° is detected and measured through Cobb method [2]. The scoliotic curve progresses during spinal growth, and is classified under three categories, according to their age of appearance, such as infantile before three years of age, juvenile between three and ten years of age (or at the beginning of puberty) and, adolescent when detected after the age of 10 or after puberty [3, 4].

Research work on twins have been performed since 1875 to observe the influence of genetic and external factors on the manifestation of certain diseases [5]. Fisher and George [6] studied pairs of monozygotic and dizygotic twins with idiopathic scoliosis, assessing the similarity of spinal deformities and observed that environmental aspects were able to modify the features and severity of the disease, especially among dizygotic twins. Kesling and Reinker [7] reported, in a systematic review, concordance rate of 73 % in 37 pairs of monozygotic twins and 36 % in 31 pairs of dizygotic twins, demonstrating greater similarity between monozygotic twins. Recently, Del Curto et al. [8] reported a pair of monozygotic twins with AIS, but with curves that diverged on type, side and, severity, suggesting that this fact would occur due to the influence of the environment or action of epigenetic factors.

The epigenetic factors are unrelated to the primary DNA sequence. Otherwise, three main mechanisms related to these changes exist: DNA methylation, modification of histones and non-coding small RNAs to modify the expression of specific genes where there is therapeutic potential [9]. Epigenetic modifications provide multicellular organisms with a system of normal gene regulation that silences portions of the genome and keeps them silent as tissues differentiate as epigenotypes: errors in this complex system from environmental and stochastic events termed epimutations can give rise to abnormal gene silencing, which may result in phenotypic variation and common disease [9, 10].

## Review

### Prevalence and genetic inheritance

In 1968, Wynne-Davies [3] conducted a study in 114 patients with idiopathic scoliosis to evaluate the familial incidence of this disease and compare it to the general population, trying to find some evidence of inheritance of the disease. In this study, they observed that 6.9 % of first-grade relatives, 3.7 % of second-grade relatives and 1.6 % of third-grade relatives were affected. With these results, they concluded that there is strong evidence of existent relationship of genetic factors with idiopathic scoliosis, along with suggestive features of dominant or polygenic inheritance.

Cowell et al. [11] studied 110 families in which 80 % of patients had AIS with other affected relatives and suggested that the appearance of isolated cases of idiopathic scoliosis is related to sporadic forms from new mutations. These authors also found high prevalence (33 %) of families affected compared to their control group from the general population (5.6 %). Czeizel et al. [12] studied 116 Hungarian families, and observed occurrence of AIS in 0.083 (±0.0037) of first-grade relatives, 0.02 (±0.0017) of second-grade relatives and 0.0084 (±0.0007) of third-grade relatives. Martin et al. [13] observed in a Spanish population that 25 % of patients had one or more relatives affected with idiopathic scoliosis in their families, and the prevalence among first-grade relatives was 5.2 % and 4.3 % in second-grade relatives. These numbers were similar to those found by Wajchenberg et al. [14] who studied the genealogy of 100 Brazilian families who had at least one individual with AIS with curves greater than or equal to 20°.

The prevalence of idiopathic scoliosis in radiographic studies, in a scholar population, ranged from 0.3 % to 15.3 %, but when curves greater than ten degrees were considered, rates dropped to values between 1.5 % and 3 %. For curves larger than 20°, prevalence was between 0.3 % and 0.5 %, and for those higher than 30°, rate was between 0.2 % and 0.3 % [4] (Table 1). The inheritance pattern for the transmission of AIS is not fully known. Multifactorial inheritance have been suggested [10], autosomal dominant [1, 3, 4, 15–17] and X-linked [11, 13, 18].

**Table 1:** Prevalence of idiopathic scoliosis [4]

Prevalence of AIS	0.3–15.3 %
Curve > 10°	1.5–3.0 %
Curve > 20°	0.3–0.5 %
Curve > 30°	0.2–0.3 %

*AIS* Adolescent Idiopathic Scoliosis

In the literature, there are several reports that AIS affects girls predominantly and that these patients have a different pattern of growth compared to unaffected girls [19–21]. When we define affected subjects as those individuals with curves higher than 15° Cobb, it has been noted that 0.3 % of boys and 1.8 % of girls between 13 and 14 years have the disease in Japan [22]. Growth and sexual maturity in girls is influenced by hereditary factors related to polymorphism of genes associated with age at menarche and stature. [23, 24].

### Etiology

The etiology of AIS remains unknown. In 1882, Adams [25] did not believe in the existence of fixed curves in the lateral deviation of the spine, suggesting that there would be primarily muscle weakness with a permissive condition of the ligaments. Currently, different factors have been suggested, as the standard deviation of growth, neuromuscular or connective tissue changes, the asymmetrical limbs and trunk growth, changes in sagittal configuration of the spine and factors related to the environment [4, 6, 7].

In 1977, Burwell & Dangerfield [26] suggested that there is some relation between the side of convexity of a thoracic scoliosis, the side of the longer upper limb, and handedness. Haderspeck & Schulz [27] have shown that imbalance of lateral bending muscle forces may increase existing scoliotic curves and it is possible that greater muscle development on the side of the dominant limb influences spinal posture in adolescents.

In 1983, Dubousset et al. [28] observed the development of scoliosis in chickens undergoing resection of the pineal gland, giving the deformity to decreased production of melatonin. This research led Dubousset et al. [29] to compare rates of melatonin to a control group, whereby they noted that scoliotic patients had 35 % lower rates overnight. However, there is still no satisfactory explanation of this observation with the evolution of the disease. It has also been observed in another study that the pinealectomyzed chickens that developed scoliosis, had increased sensitive somatosensory evoked potential latency, suggesting that the lesion would affect the sensory conduction at the ceiling of the third ventricle [30].

Anatomical alterations of neural tissue, observed by MRI, were also attached to the etiology of idiopathic scoliosis. The presence of syringomyelia in the cervicothoracic region, associated with a type I Chiari malformation, in the foramen magnum had a higher prevalence in patients with idiopathic scoliosis. In 1998, Freitas et al. [31] reported that 14.5 % of the patients in a Brazilian population had this associated finding [31], while Gupta et al. [32] in the same year, through literature review, found this type of variations between 17 and 47 % [32]. Studies show that patients generally have no neurological disorders, detecting in some patients, abdominal reflex asymmetry [33]. However, up to date it is unknown whether this finding is a cause or consequence of the deformity.

Royo-Salvador et al. [34] proposed that spinal cord traction caused by a tight filum terminale may be considered as a pathogenic mechanism involved in the development of syringomyelia, Chiari malformation (Type 1) and scoliosis. These authors performed ressection of the filum terminale through a standard sacrectomy in 20 non-consecutive patients with scoliosis (n = 8), syringomielia (n = 5), Chiari malformation (n = 2) and a combination of all three conditions (n = 5). All patients were neurological symptomatic. Improvement of the scoliotic curves was observed in all patients with scoliosis except in one. In this paper the authors do not reveal the etiology of scoliosis.

Relative anterior spinal overgrowth with uncoupled neuro-osseous growth was also found in AIS girls. The observations are closely related to bone and energetic metabolism. Burwell et al. [35] have suggested the possible role of leptin dysfunction in abnormal asymmetric growth that leads to spinal deformity. The effect of leptin and soluble leptin receptor (sOB-R) on bone and energetic metabolism matches the phenotypic expression of the AIS girls. In 2012, Liu et al. [36] observed that the sOB-R level in AIS girls was significantly higher than that of healthy control girls in the absence of difference in free serum leptin level.

Some authors have suggested that AIS could be due to lesion in the anterior horn of the spinal cord or in the brain stem since type grouping was detected examining spinal muscles [37, 38]. In 1998, Chagas et al. [39] reported that the neurogenic pattern is the main manifestation found in the rotator muscles of the back of 32 female patients, biopsied during AIS correction surgery and sent to histochemical study analysis. These authors suggested the presence of a smooth form of type III spinal muscular atrophy [39]. In 2015, in a similar study, Wajchenberg et al. [40] found peri and endomysial fibrosis, fatty proliferation between muscle fibers and signs of “Central Core”, both at the concavity and the convexity of the curve, suggesting that the AIS is a congenital myopathy.

### Genetic research

Since 2000, studies of genetic link in families with multiple affected members suggested possible chromosomal regions related to the etiology of AIS (Table 2). Using this technique, Wise et al. [14] in 2000, described the first regions related to AIS, on chromosomes 6p, 10q and 18q, after studying a large family with seven members affected by this disease. In the following years, other family studies have suggested that adolescent idiopathic scoliosis were related to chromosomes 6, 9, 16 and 17 [41], 17p11 [42], 19p13.37 [43], 8q12 [44], 9q31-q34.2 and 17q25.3-qtel [1], 12p23 [45] and 18q12.1-12.2 [46]. Wajchenberg et al. [47] found no connection when studied a Brazilian family with multiple affected members.

**Table 2:** Genetic research that suggested possible chromosomal regions related to the etiology of adolescent idiopathic scoliosis

Wise et al., 2000 [16]	6p, 10q, 18q
Chan et al., 2002 [43]	19p13.37
Miller et al., 2005 [41]	6, 9, 16, 17
Gao et al., 2007 [44]	8q12
Ocaka et al., 2008 [1]	9q31-q34.2, 17q25.3-qtel
Raggio et al., 2009 [45]	12p23
Gurnett et al., 2009 [46]	18q12.1-12.2
Clough et al., 2010 [42]	17p11
Takahashi et al., 2011 [50]	10q24.31
Kou et al., 2013 [53]	6q24.1
Zhu et al., 2015 [54]	1p36.32, 2q36.1, 18q21.33, 10q24.32

Due to the higher prevalence of AIS in girls, studies were conducted to analyze the sex chromosome X. In 2002 Inoue et al. [48] reported that the polymorphism of estrogen receptor gene is associated with the severity of the deformity. Justice et al. used independent analysis model with 15 markers for the X chromosome in 202 families, finding association in the regions Xq23 and Xq26.1 [18]. Wu et al. [49], investigated the association of the polymorphism of estrogen receptor gene and they concluded that the AIS is associated to XbaI gene, however Takahashi et al. [50] in 2011 reported no relationship between the two regions at the chromosome X, related to the estrogen receptor genes, with the manifestation and severity of AIS, after performing an extensive study in the Japanese population.

Also in 2011, Takahashi et al. [51] in a multicenter study conducted in the Japanese population with 1050 women with AIS and 1474 controls, using the genome wide association study (GWAS) technique, associated the loci 10q24.31 to the disease. This region contains the LBX1 gene, which is expressed in the dorsal region of the spinal cord, skeletal muscle and influence somatosensitive neuronal activity. Family studies to search for genes responsible for AIS can help us to understand the origin of the disease, but have an important limitation to separate affected individuals to those not affected because there is variability in the measurement of the curves using Cobb’s method and even in clinical evaluation [52].

In 2013, Kou at al. [53] extended the Takahashi et al. [51] study. Not only did this extension confirmed the relationship of 10q24.31 with AIS but it also identified a new correlated DNA region, region 6q24.1, and related a Han Chinese population to another population of European ancestry. The authors also reported a relationship between the disease and GPR 126 gene within the same region studied. According to the authors, neither the expression of this gene in the skeletal tissue of humans nor its relationship to scoliosis has been explored. Thus, they attempted to study its expression in various tissues, such as bone, cartilage, intervertebral disks, and they found high expression in cartilage.

Recently, Zhu et al. [54] conducted a research in a Chinese Han population using GWAS and identified three new susceptibility loci of AIS at 1p36.32, 2q36.1 and 18q21.33. The authors also refined a region previously associated with AIS at 10q24.32, suggesting that LBX1AX1, an antisense transcript of LBX1, might be the functional variant of AIS [54].

Thus, another alternative for genetic studies is the use of molecular markers, previously selected and with a known location in the human genome such as SNPs or snips, which are “silent” chromosomal regions, easily located, without phenotypic manifestation and that show variability of heterozygous alleles. Authors like Aulisa et al. [55], Quiu et al. [56], Chen et al. [57] and Wajchenberg et al. [58], carried out studies of genetic polymorphisms trying to relate AIS with changes in certain genes.

Current statistics show that AIS is responsible for 90 % of cases of idiopathic scoliosis, reaching more than 2 % of the pediatric population, providing more than 600,000 medical visits per year [59]. Due to these numbers studies were further conducted, using a microarray technique, in long series of patients with AIS, enabling the discovery of several markers of polymorphism in chromosome regions related not only to the disease but also to its progression, through a 1 to 200 scale [59]. However, this method has high cost, was developed only for the Caucasian population with no validation for African and Asian populations and needs further confirmation in the literature.

We, like Cheng et al. [60] believe in the need for more cross-disciplinary and multicenter research collaboration to enable pooling of clinical cases to facilitate cross-ethnic studies with sufficient numbers of patients to better define phenotypes and endophenotypes. This approach would also enable better complementary use of expertise and emerging innovative technologies and, along with wiser use of advances in other fields, could lead to improvements in research methodology and in understanding disease trajectory [60].

## Conclusion

AIS is a complex disease and, for the most part, complex diseases are caused by a combination of genetic, environmental, and lifestyle factors, most of which have not yet been identified [61]. Despite all studies and technology, we still can not fully describe their way of inheritance and which are the responsible genes. Family studies may assist to understand the disease, but the great challenge is to label which individuals are affected since there is great variability in the presentation, being doubtful to consider who is an affected individual just by measuring the deformity by the Cobb method. One should also consider that certain groups are more susceptible, such as females and Caucasians. This is probably a polygenic disease and influenced by genes responsible for the synthesis of proteins important for the composition of human tissues, which act in supporting the spine. Thus, it becomes important to study the polymorphism of these genes and their expression in tissues, also considering the influence of the environment.

## Abbreviations

AIS: Adolescent idiopathic scoliosis
GWAS: Genome wide association study
sOB-R: Soluble leptin receptor
